# Emerging Biomarker-Guided Therapies in Prostate Cancer

**DOI:** 10.3390/curroncol29070400

**Published:** 2022-07-18

**Authors:** Jasna E. Deluce, Luisa Cardenas, Aly-Khan Lalani, Saman Maleki Vareki, Ricardo Fernandes

**Affiliations:** 1 Division of Medical Oncology, Department of Oncology, Schulich School of Medicine & Dentistry, London Health Sciences Centre, Western University, London, ON N6A 5W9, Canada; jasna.deluce@lhsc.on.ca; 2 Juravinski Cancer Centre, McMaster University, Hamilton, ON L8V 5C2, Canada; cardenas@hhsc.ca (L.C.); lalania@hhsc.ca (A.-K.L.); 3 Division of Experimental Oncology, Department of Oncology, Schulich School of Medicine & Dentistry, Western University, London, ON N6A 5W9, Canada; 4 Department of Pathology and Laboratory Medicine, Schulich School of Medicine & Dentistry, Western University, London, ON N6A 5C1, Canada; 5 Cancer Research Laboratory Program, Lawson Health Research Institute, London, ON N6C 2R5, Canada

**Keywords:** prostate cancer, predictive biomarkers, immunotherapy, theranostics, PARP inhibitors

## Abstract

Prostate cancer remains one of the leading causes of cancer death in men worldwide. In the past decade, several new treatments for advanced prostate cancer have been approved. With a wide variety of available drugs, including cytotoxic agents, androgen receptor axis-targeted therapies, and alpha-emitting radiation therapy, identifying their optimal sequencing remains a challenge. Progress in the understanding of the biology of prostate cancer has provided an opportunity for a more refined and personalized treatment selection process. With the advancement of molecular sequencing techniques, genomic precision through the identification of potential treatment targets and predictive biomarkers has been rapidly evolving. In this review, we discussed biomarker-driven treatments for advanced prostate cancer. First, we presented predictive biomarkers for established, global standard treatments for advanced diseases, such as chemotherapy and androgen receptor axis-targeted agents. We also discussed targeted agents with recent approval for special populations, such as poly ADP ribose polymerase (PARP) inhibitors in patients with metastatic castrate-resistant prostate cancer with homologous recombination repair-deficient tumors, pembrolizumab in patients with high levels of microsatellite instability or high tumor mutational burden, and prostate-specific membrane antigen (PSMA) directed radioligand theragnostic treatment for PSMA expressing tumors. Additionally, we discussed evolving treatments, such as cancer vaccines, chimeric antigen receptor T-cells (CAR-T), Bispecific T-cell engagers (BiTEs), other targeted agents such as AKT inhibitors, and various combination treatments. In summary, advances in molecular genetics have begun to propel personalized medicine forward in the management of advanced prostate cancer, allowing for a more precise, biomarker-driven treatment selection with the goal of improving overall efficacy.

## 1. Introduction

**Figure 1:**
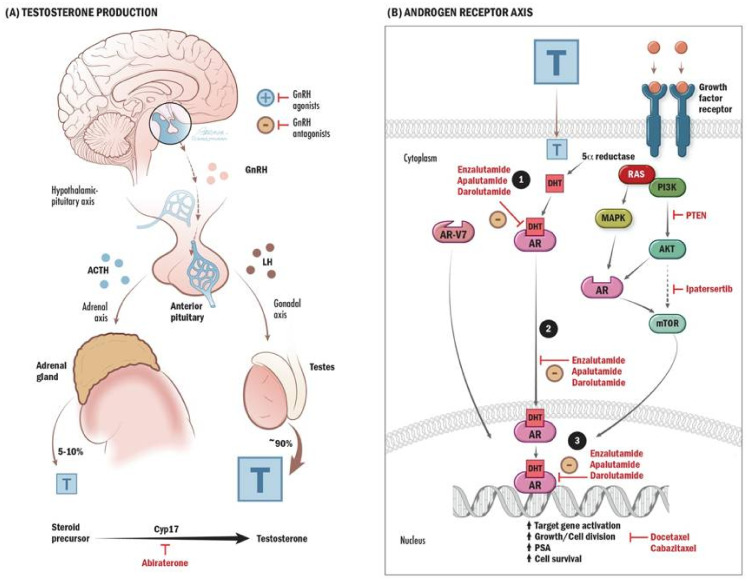
Androgen Synthesis and Mechanism of Action. (**A**) Gonadotropin-releasing hormone (GnRH) is typically released from the hypothalamus in a pulsatile fashion to signal the anterior pituitary to release follicle-stimulating hormone (FSH) and luteinizing hormone (LH), which then signal the gonads to produce testosterone through the hypothalamic–pituitary–gonadal (HPG) axis and is responsible for 90% of the body’s testosterone production (represented by T in the figure). GnRH also signals the pituitary to release adrenocorticotropin-releasing hormone (ACTH) to signal the adrenal glands to produce androgens. Chemical androgen deprivation therapy (ADT) consists of GnRH agonists or antagonists, which inhibit these pathways. The production of testosterone itself involves the conversion of steroid precursor molecules into testosterone in part by the enzyme cyp17. Abiraterone is an androgen receptor-axis targeted agent (ARAT) that works by inhibiting this enzyme and, ultimately, androgen production. (**B**) Testosterone, as steroid molecules, typically then diffuses through the cell membrane into the prostate cancer cell, where they are converted by 5-alpha-reductase into dihydrotestosterone (DHT), which then binds to the intracellular androgen receptor (AR). This receptor complex then migrates into the nucleus and is involved in transcription and translation of genes involved in cell growth and division. Chemotherapy such as docetaxel and cabazitaxel exert their effects by interfering with the cell cycle, growth, and division. Enzalutamide, Apalutamide, and Daralutamide are ARATS that work by inhibiting binding of DHT to AR, translocation to the nucleus, and transcription of genes. Acquired resistance to this pathway can develop when other mutations occur. When the androgen receptor is mutated, such as the AR-V7 splice variant, it is able to exert its effects on cell growth independent of androgen binding and is also resistant to binding of competitive inhibitors such as enzalutamide. The androgen receptor pathway is also inversely related to the PI3K/AKT/mTOR pathway, such that inhibition of one often leads to upregulation of the other via acquired mutations. PTEN and mTOR inhibitors were investigated as possible drug targets for prostate cancer. The AKT inhibitor, Ipatasertib, showed some activity and potential benefit in prostate cancer in combination with abiraterone.

Prostate cancer remains the most common malignancy in men, with an incidence of 11.3 per 100,000 men per year in the United States (2014–2018), and is the fifth most common cause of cancer-related mortality with 18.9 deaths per 100,000 (2015–2019) [1]. While most prostate cancers are detected at early, curable stages, advanced prostate cancer remains largely incurable. The 5-year overall survival for prostate cancer patients with distant metastases was 30.6% (2011–2017) [1]. Currently, the backbone treatment for non-metastatic castrate-resistant prostate cancer (nmCRPC), metastatic castrate-sensitive prostate cancer (mCSPC) and metastatic castrate-resistant prostate cancer (mCRPC) remains androgen-deprivation therapy (ADT) with either surgical castration via bilateral orchiectomy, or chemical castration with luteinizing hormone-releasing hormone (LHRH) agonists/antagonists [2,3]. LHRH agonists and antagonists interrupt the hypothalamic–pituitary–gonadal axis signaling by inhibiting luteinizing hormone (LH) release, leading to decreased production of androgens by the testes and thus cell cycle arrest of the prostate cancer cells (Figure 1A) [2].

In addition to ADT, there are several treatments with various mechanisms of action that were proven effective in the treatment of prostate cancer when added to ADT, including androgen receptor axis-targeted agents (ARATs), chemotherapy, targeted therapies, alpha emitter radium 223, immunotherapy, and more recently, theranostics [4]. Despite these options being available, there is little evidence for optimal sequencing of treatments to maximize survival while minimizing the risk of toxicities. With the advent of molecular sequencing, liquid biopsies, and advanced imaging techniques, evolving predictive biomarkers are being investigated and may become future drivers of clinical decision-making for a more precise, biomarker-based selection of treatments in this space [5]. Next-generation sequencing tests were used to isolate circulating tumor cells (CTCs), cell-free deoxyribonucleic acid (cfDNA), and circulating tumor DNA (ctDNA). cfDNA consists of small nuclear acid fragments in the bloodstream as a result of apoptosis or necrosis of any cell in the body, whereas more specifically, ctDNA is a component of these fragments that are released specifically from the lysis of tumor cells. Circulating tumor cells (CTCs), on the other hand, consist of released intact tumor cells, which are lysed in the bloodstream [6]. ctDNA and CTCs were used to identify molecular mutations in cancers that can be used to prognosticate or predict response to treatment in prostate cancer [7]. CTCs and ctDNA can be isolated from the plasma of patients with mCRPC to identify mutations in oncogenes such as BRCA1, BRCA2, and ATM genes [8]. Actionable mutations involving the phosphatidylinositol-3-kinase (PI3K) pathway were also detected upon disease progression [9].

With the large variety of available treatments for advanced prostate cancer, it is important for clinicians to access tools that will aid in the selection of highly effective therapies with the least risk of toxicity for a specific patient, and advances in molecular sequencing and the use of predictive biomarkers may achieve that [4]. Current challenges include translating the heterogeneity of prostate cancer to individualized treatment, identifying and utilizing biomarkers that predict survival and/or treatment response, as well as designing optimal tools to help guide precision medicine.

Throughout this review, we discussed the current treatment landscape with various approved and investigational biomarker-driven therapies for advanced prostate cancer and their predictive biomarkers.

## 2. Methods

A non-systematic literature search was conducted on PubMed using the terms ‘advanced prostate cancer’, ‘predictive biomarkers’, ‘precision oncology’, ‘PARP inhibitors’, ‘Immunotherapy’, ‘AKT inhibitors’, ‘PI3K’, ‘PTEN’, ‘PROTACS’, ‘vaccines’, and ‘Theranostics’. The same search terms were used for the ClinicalTrials.gov registry of clinical trials. Review papers and clinical trials were retrieved and published between the years 2015 and 2022. Additional articles were retrieved based on the content of the initial articles reviewed, and additional searches were conducted, including search terms ‘PSMA’, ‘PDL-1’, ‘ERG-SOX9’, ‘Serum testosterone’, ‘HRR’, ‘TMB’, and ‘MSI’ in combination with ‘advanced prostate cancer’ and/or ‘predictive biomarkers’. Only English studies were included.

### 2.1. Androgen Receptor Axis-Targeted Agents (ARATs)

Prostate cancer cells eventually develop resistance to ADT alone as the tumor becomes castration-resistant. While ADT works by inhibiting the production of androgens by the testis by interfering with the hypothalamic–pituitary–gonadal axis, a small portion of androgens can also be produced intratumorally or by the adrenal glands or other peripheral tissues [10]. Furthermore, resistance can occur when androgen receptors are upregulated. ARATs were thus developed to try and overcome some of this resistance.

ARATs include abiraterone acetate with prednisone, enzalutamide, darolutamide, and apalutamide and are used in addition to ADT to treat nmCRPC (enzalutamide, darolutamide, apalutamide), mCSPC (abiraterone, enzalutamide, and apalutamide), and mCRPC (abiraterone and enzalutamide), regardless of prior chemotherapy [11,12,13,14,15,16,17,18,19,20,21,22,23]. Abiraterone is a cytochrome P450 family 17 subfamily A member 1 (CYP17A1) inhibitor, used in combination with prednisone (to avoid toxicity), which decreases the production of androgens by interfering with androgen synthesis (Figure 1A) [24]. CYP17A1 is an enzyme involved in the conversion of cholesterol steroid precursors into testosterone. By inhibiting this enzyme, testosterone cannot be synthesized by this pathway. By contrast, enzalutamide, apalutamide, and darolutamide all act as competitive inhibitors of the androgen receptor, preventing activation of the receptor, translocation to the nucleus, and transcription of downstream genes (Figure 1B) [15,25]. Clinically, ARATs are often preferred over chemotherapy as a first-line treatment due to a more favorable toxicity profile and ease of oral administration, unless there is particularly aggressive histopathology or high volume, symptomatic disease, where there may be an impending visceral crisis that would warrant choosing a therapy with rapid response rate. Despite this, optimal sequencing between chemotherapy and ARATs has not clearly been determined. Head-to-head studies comparing ARATs to chemotherapy tend to be retrospective, which is limited by selection bias without the benefit of randomization [26].

#### 2.1.1. Neutrophil-to-Lymphocyte Ratio

The neutrophil-to-lymphocyte (NLR) ratio is a biomarker calculated from routine bloodwork that is a prognostic indicator for a number of malignancies. Interestingly, a retrospective study found that in patients with nmCRPC and mCRPC NLR of less than 2.5 was associated with better cancer-specific survival (*p* = 0.026) and radiographic progression-free survival (rPFS; *p* = 0.011) when they received docetaxel prior to ARATs (enzalutamide or abiraterone) rather than ARATs followed by docetaxel [27]. Sequencing did not seem to alter outcomes for patients with NLR of greater than 2.5 [27]. This study suggests that NLR could be a useful biomarker to predict optimal sequencing of treatment in the castrate-resistant setting if NLR is less than 2.5.

#### 2.1.2. Serum Testosterone

Low levels of testosterone at baseline could be predictive of response to ARATs. Pre-treatment serum testosterone levels below 5 ng/dL had a longer duration of prostate-specific antigen (PSA) response in patients treated with abiraterone compared to enzalutamide in CRPC (62% vs. 32%; *p* = 0.033) [28]. This would suggest potentially choosing abiraterone over enzalutamide if baseline serum testosterone is low. In a similar study, patients with pre-treatment testosterone levels greater than 0.05 ng/mL treated with enzalutamide had improved PFS (*p* = 0.047) and a trend towards improved overall survival (OS; *p* = 0.18) compared to patients with pre-treatment testosterone levels less than 0.05 ng/mL, while the opposite pattern was shown for docetaxel [29]. Presumably, cancer that grows despite a low testosterone environment is likely to be more resistant to androgen receptor-targeted treatments and potentially more susceptible to cytotoxic drugs.

#### 2.1.3. Androgen Receptor Splice Variant 7

Androgen receptor splice variant 7 (AR-V7) is a mutation that results in an isoform of the androgen receptor that alters its ligand-binding domain that binds to androgens or to their competitive inhibitors and allows the receptor to remain in an active state independent of androgen ligand binding (Figure 1B) [30]. AR-V7 splice variants mRNA can be detected in CTCs, and are a predictive biomarker, as the change in binding domain site results in resistance to ARAT but not taxanes [31]. Typically, the physician’s choice between ARAT and chemotherapy is driven by clinical factors such as disease burden and visceral involvement; however, genetic testing for the AR-V7 splice mutation in tumor tissue could potentially be a helpful biomarker as treatment with taxanes resulted in improved OS and PFS compared to abiraterone or enzalutamide in patients with this mutation [31,32].

In one study, AR-V7 protein expression via immunohistochemistry was determined for 358 primary prostate samples and 293 metastatic biopsies, and outcomes including disease progression, response, and gene expression were determined [33]. Authors found that AR-V7 was expressed in less than 1% of primary prostate cancer but significantly increased in expression following treatment with abiraterone or enzalutamide (*p* = 0.020), suggesting the mutation may develop as acquired resistance to these drugs [33]. Furthermore, AR-V7 negative tumors were associated with better PSA response (100% vs. 54%; *p* = 0.03) and overall survival (74.3 vs. 25.2 months, HR 0.23 [0.07–0.79]; *p* = 0.02) from endocrine therapies [33]. Thus, the presence of AR-V7 is a negative predictor of response to endocrine therapy. Interestingly, the study also showed that AR-V7 expression was heterogenous between metastases sites within the same patient, making it a challenge to reliable utilize as a biomarker [33]. Furthermore, tissue samples are often obtained at the time of initial diagnosis, which would miss any mutations that evolved as acquired resistance and necessitate the need for further tissue sampling on progression with invasive biopsies.

#### 2.1.4. Circulating Tumor Cells, Cell-Free DNA, and Circulating Tumor DNA

Liquid biopsies from plasma samples have emerged as a complementary method to traditional tissue biopsy for diagnostic, predictive, and prognostic value [34]. The benefit is that they are less invasive and can easily be obtained at various states of prostate cancer on progression, overcoming the barriers of traditional tissue biopsy. CTCs are whole in-tact tumor cells shed from a primary or metastatic tumor into the peripheral blood and lymph system [34]. Messenger ribonucleic acid (mRNA) from these cells can be detected by reverse-transcriptase polymerase chain reaction (RT-PCR) and can be utilized to detect mutations such as AR-V7. As an example, one study used quantitative RT-PCR to detect AR-V7 in CTCs from patients with mCRPC initiating treatment with abiraterone or enzalutamide and followed them prospectively for a response. In men receiving enzalutamide, participants with AR-V7 positive tumors in CTCs had lower PSA response rates (0%) versus participants with AR-V7 negative tumors (53%; *p* = 0.004), as well as shorter PSA PFS (median 1.4 months vs. 6.0 months; *p* < 0.001), clinical or radiographic PFS (median 2.1 months vs. 6.1 months; *p* < 0.001) and OS (median 5.5 months vs. not reached; *p* = 0.002) [32]. In participants who received abiraterone, similar results were reported such that participants with AR-V7 positive tumors had lower response rates than AR-V7 negative tumors (0% vs. 68%; *p* = 0.004), shorter PSA PFS (median 1.3 months vs. not reached; *p* < 0.001), clinical or radiographic PFS (median 2.3 months vs. not reached *p* < 0.001) and OS (median 10.6 months vs. not reached; *p* = 0.006) [32]. Thus, the presence of AR-V7 in CTCs is a negative predictor of response to ARATs. Furthermore, a similar study found that clinical outcomes were superior with taxanes compared to enzalutamide or abiraterone in patients with mCRPC in AR-V7 positive tumors, whereas outcomes did not differ by treatment type if the tumor was AR-V7 negative and thus, AR-V7 mutation is not associated with resistance to taxanes [35].

cfDNA in the bloodstream is made up of both ctDNA and somatic cell DNA fragments released by cells undergoing apoptosis or necrosis. In a cross-sectional cohort study at Memorial Sloan Kettering Cancer Center, 161 men with progressive mCRPC undergoing a change in the treatment had their blood analyzed and were followed for 3 years [31]. The results showed that AR-V7 ctDNA was found in 18% of samples. Patients whose blood samples showed AR-V7-positive circulating tumor DNA (ctDNA) before use of ARAT had shorter rPFS (median 2.3 vs. 14.5 months; *p* < 0.001), shorter time on therapy (median 2.1 vs. 6.8 months; *p* < 0.001), and shorter OS (median 4.6 months vs. not reached; *p* < 0.00) than those without the AR-V7-positive ctDNA. A multivariable model adjusting for baseline factors associated with survival also showed superior OS with taxanes relative to ARAT when AR-V7-positive ctDNA were detected prior to therapy (HR = 0.24; 95% CI 0.10–0.57; *p* = 0.035) [31]. New drugs are currently being investigated which are non-competitive inhibitors of the AR and could, in theory, overcome the resistance of AR-V7 variants as the drug binds to an alternative binding site [36].

Although AR amplification and gene mutations that are detectable in ctDNA and cfDNA from mCRPC were identified as negative predictors of response to treatment from enzalutamide and abiraterone, this testing is not typically utilized in the first line for this indication [8]. AR-V7 is both a predictive and a prognostic biomarker, making interpretation of results challenging as the prognostic value is confounded [26]. Furthermore, although it may be possible for a patient’s tumor to be AR-V7 negative on progression after enzalutamide or abiraterone, some evidence suggests that cabazitaxel should be chosen as a subsequent line of treatment over the other ARAT (either enzalutamide or abiraterone, whichever had not been previously used) in the setting of prior taxane use [37]. In this instance, the absence of the AR-V7 mutation should not necessarily mean that switching therapy to another ARAT is the optimal sequencing. However, AR-V7 may still be useful as a biomarker for treatment decision-making as many patients and clinicians tend to avoid chemotherapy when possible due to concerns with toxicity in a population that is often older with significant comorbidities [26]. Liquid biopsies for cfDNA or ctDNA are proving to be effective tools to overcome the difficulty and invasive nature of traditional biopsies, as obtaining blood samples is non-invasive and generally has adequate concordance with tissue biopsy, albeit slightly less sensitive [26]. ctDNA can also identify mutations in oncogenes with actionable treatment targets (discussed throughout the remainder of this manuscript), such as BRCA1, BRCA2, and ATM genes, as well as the PI3K pathway genes [8,9].

### 2.2. Chemotherapy

#### 2.2.1. Taxanes

Taxane chemotherapy works by inhibiting microtubule depolymerization, causing cell cycle arrest (Figure 1B) [38,39]. Docetaxel is approved in both the mCSPC and mCRPC settings, is particularly effective in the treatment of high-volume disease, and is often the drug of choice when disease progression is rapid or there is an impending visceral crisis [40,41]. Cabazitaxel is approved in the mCRPC setting for patients previously treated with docetaxel [42].

##### Low Serum Testosterone

As described in the previous section on ARATs, treatment with taxane chemotherapy may be more effective in patients with low pre-treatment testosterone levels [29]. Although the underlying mechanism for this phenomenon is unclear, it may be because more resistant tumors are likely to grow despite an environment with low levels of testosterone. Some research points to the presence of prostate cancer itself being the cause of low serum testosterone by way of secreting proteins that lead to negative feedback and suppression of the hypothalamic–pituitary–gonadal axis [43]. Translational research in mice, however, has shown that prolonged exposure to low testosterone environments may induce selective pressures to upregulated oncogenes or tumor mutagenesis that favors more aggressive, hormone-refractory tumors [44]. Therefore, such prostate cancers would potentially be of higher grade or may contain more rapidly dividing tumor cells, which would then be more susceptible to treatment that disrupts cell division, and more resistant to treatment that aims to inhibit the androgen pathways.

##### ERG/SOX9

There are also emerging biomarkers that are negative predictors of response to taxane treatment. E26 transformation-specific (ETS)-related gene (ERG) is an oncogene overexpressed in prostate cancer, along with its downstream effector SRY-related HMG box (SOX)-9 [45]. ERG binds to microtubules inhibiting their interaction with docetaxel, thus leading to innate resistance [45]. Seventy-nine patients with mCRPC treated with docetaxel were evaluated with immunohistochemistry, and treatment outcomes were assessed. The results showed that patients with positive immunohistochemistry for ERG and SOX9 had lower PSA response (ERG-positive 15.4% vs. 62.1%, *p* = 0.004; SOX9 46.8% vs. 100.0%, *p* = 0.003) and shorter PFS (ERG positive PSA-PFS 3.2 months vs. 7.4 months, *p* < 0.001, clinical/radiographic-PFS 3.8 months vs. 9.0 months, *p* < 0.001 and OS 10.8 months vs. 21.4 months, *p* < 0.001; SOX9 positive PSA-PFS, clinical/radiographic-PFS and OS (*p* = 0.006, *p* = 0.012 and *p* = 0.023, respectively) following treatment with docetaxel [45]. Similar results were observed in a study examining ERG expression in peripheral blood using quantitative real-time polymerase chain reaction for patients with mCRPC treated with taxane therapy [46].

#### 2.2.2. Platinums

Platinum chemotherapy forms covalent links within DNA structure, causing catastrophic DNA damage and cell death. Aggressive-variant prostate cancers are often AR-independent and treatment-refractory, associated with poor prognosis, and are often tumors with neuroendocrine features (NEPC) [26]. These cancers are often unlikely to respond to ARAT treatment but do show a high response rate -although short in duration- to platinum-based chemotherapy [47,48]. Evidence is extrapolated from the treatment of small cell lung cancer, and thus, platinum doublet therapy is the preferred therapy of choice [49]. Aside from the current standard utility of platinum-based therapy in rare variant/small-cell histologies of the prostate, platinum agents have not been effective in improving OS in most studies of prostate cancer [50].

##### DNA Repair Genes

Interestingly, however, Mota et al., evaluated the response to platinum-based chemotherapy in patients with mutations in DNA repair genes, including BRCA1, BRCA2, AATM, PALB2, CDK12, and FANCA in taxane-refractory patients and observed an increased likelihood in PSA response of more than 50% compared to patients who lacked such genetic alterations (OR 7.0; 95% CI 1.9 to 29.2), although there was no difference in OS [51].

##### SLFN11

Similarly, high expression levels of the DNA/ribonucleic acid (RNA) helicase Schlafen family member-11 (SLFN11) gene detected in mCRPC patients by liquid or tissue biopsy were associated with longer rPFS (6.9 vs. 2.8 months, HR = 3.72; 95% CI 1.56–8.87; *p* < 0.001) and PSA decline. The PSA decline was greater than 50% in all patients who were treated with platinum-based chemotherapy. However, there was no difference seen in OS regardless of SLFN11 expression [52].

### 2.3. Radiopharmaceuticals

#### Radium-223

Radium-223 is an alpha-emitting radioactive agent that targets bone metastases in prostate cancer, and provides OS benefit as well as palliation of pain (median, 14.9 months vs. 11.3 months; hazard ratio, 0.70; 95% CI, 0.58 to 0.83; *p* < 0.001) [53]. Beta-emitting agents, including strontium-89 or Samarium-153, seem to palliate symptomatic bony metastases but did not confer an OS benefit [54]. Although some studies show a change in bone metabolic markers during treatment with radium-223, such as N-telopeptide and bone-specific alkaline phosphatase, which may correlate with response to treatment and survival, they may be more important prognostically than as predictive biomarkers to drive clinical decision making [55,56].

### 2.4. PSMA-Directed Therapy

#### Prostate-Specific Membrane Antigen (PSMA)

**Figure 2:**
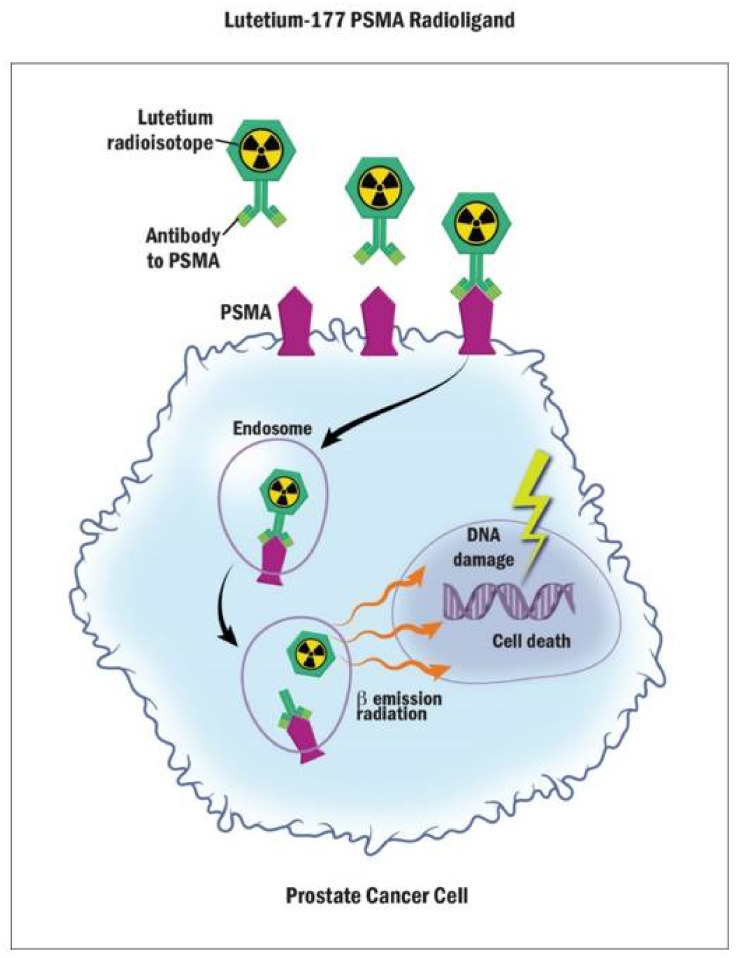
Mechanism of Action of Lutetium-177 PSMA Radioligand.

PSMA is a cell surface receptor that is specific to prostate tissue and is found to be overexpressed in some metastatic prostate tumors [57]. When PSMA binds its ligand, the receptor internalizes the ligand into endosomes [58]. Lutetium-177 (177Lu)-PSMA radionuclide therapy was recently shown to have a survival benefit in mCRPC after progression on an ARAT and at least one taxane [59]. 177Lu-PSMA is a beta-emitting radioligand bound to an antibody or small peptide that binds to PSMA, such that the radioisotope is then targeted directly to PSMA expressing tissue [60]. The radioisotope conjugate then binds to PSMA and is internalized into the cell via endosomes, thus concentrating the radioisotope in PSMA-expressing prostate tissue while sparing most healthy tissues (Figure 2) [60].

^177^-Lu-PSMA-617 is a beta-emitting radioligand bound to a PSMA antibody or small peptide that attaches to PSMA. When the antibody binds PSMA, the whole complex is engulfed in the cell via endosomes, whereby the ^177^-Lu radioisotope is then released, causing damage to the cell DNA in the nucleus and radiation to surrounding healthy cells.

In one phase II study, patients underwent a Gallium-68 (^68^Ga)-PSMA-11 positron emission tomography (PET)/computed tomography (CT) scan, which detects PSMA expression, and mean and maximum standardized uptake values (SUV) were recorded [61]. Higher mean SUV and maximum SUV intensity levels were associated with response to treatment with 177Lu-PSMA-617 treatment as measured by the decline in PSA levels [61]. Responders had a maximum SUV of 44 (±5), compared to 17 (±9) in non-responders (*p* < 0.07), and a mean SUV of 10 (±4) vs. 6 (±4) (*p* < 0.04) [61]. Interestingly, patients that had a maximum SUV of less than 15 on PSMA-PET did not show a significant response with PSA decline [61]. More recently, the VISION trial was the first phase III randomized controlled trial evaluating ^177^Lu-PSMA-617 in addition to standard of care (excluding chemotherapy, immunotherapy, and radium-223) vs. standard of care alone and showed improved rPFS (median 8.7 vs. 3.4 months; hazard ratio for progression or death, 0.40; 99.2% confidence interval [CI], 0.29 to 0.57; *p* < 0.001) and OS (median, 15.3 vs. 11.3 months; hazard ratio for death, 0.62; 95% CI, 0.52 to 0.74; *p* < 0.001) [59]. Alpha radioligand PSMA-targeted treatment such as actinium (^225^Ac-PSMA-617) was also investigated, as it has higher anti-tumor activity; however, toxicity is also higher [62]. One limitation to the use of theragnostic PSMA-targeted treatment is that PSMA is also expressed in small amounts in some healthy tissues, including the kidney and salivary glands; therefore, radiotoxicity can be problematic [63]. Additionally, not all prostate cancers are PSMA-PET avid, limiting the utility of treatment in non-PSMA expressing tumors.

Bispecific T-cell-engager molecules (BiTE) are another type of PSMA-directed therapy, whereby it has binding domains to both PSMA as well as CD3 on T-cells as a way of engaging T-cells and delivering them directly to PSMA-expressing tissue resulting in cytotoxic tumor cell killing [64]. Pasotuxizumab was shown to have some anti-tumor activity in combination with pembrolizumab in a recent phase I dose-escalation trial [65,66]. Chimeric antigen receptor (CAR) T-cells are another PSMA-targeted immunotherapy under investigation for prostate cancer. CAR-T works by modifying a patient’s own T-cells and engineering them to target PSMA to essentially deliver immune cells to the tumor [5]. When combined with other immune-based therapies such as Interleukin-2, it presents a promising treatment to explore [67,68].

### 2.5. Targeted Therapy

#### 2.5.1. Poly (ADP-Ribose) Polymerase (PARP) Inhibitors

**Figure 3:**
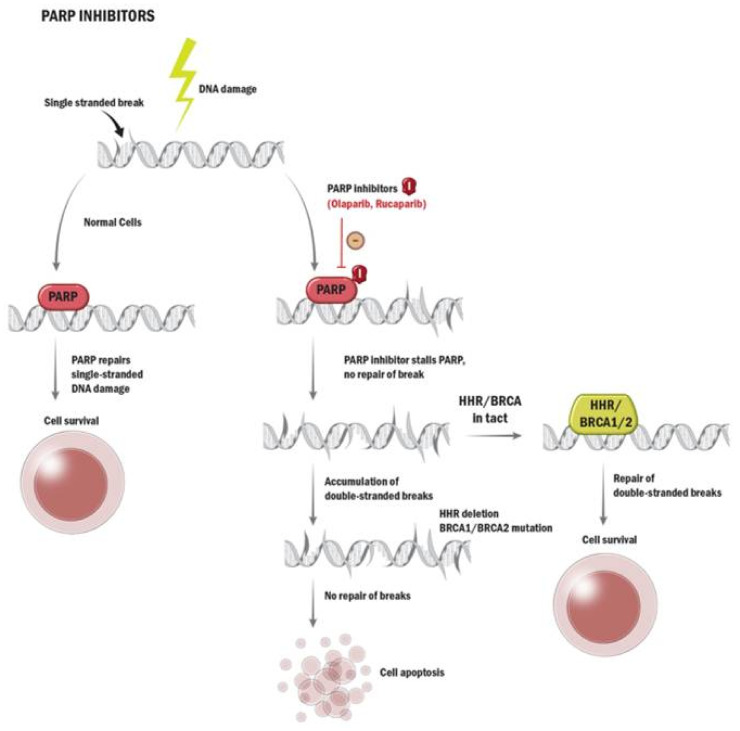
Mechanism of action of PARP inhibitors. Poly (ADP-ribose) polymerase (PARP) is a protein involved in repair of single-stranded DNA breaks. When inhibited by a PARP inhibitor drug (represented by a red oval with the letter “I”), the PARP protein stalls at its attachment point on DNA strands. This lack of repair of single-stranded breaks leads to accumulation of double-stranded breaks. In patients who also lack double-stranded repair mechanisms, such as those with homologous recombination repair (HRR) gene mutations, this leads to synthetic lethality and cell death.

Poly (ADP-ribose) polymerase (PARP) inhibitors, such as olaparib and rucaparib, are oral targeted treatments approved for use in patients with mCRPC. PARP is a protein involved in the repair of single-stranded DNA breaks [69]. When inhibited, the lack of repair leads to the accumulation of double-stranded breaks [69]. In patients who lack double-stranded break repair mechanisms, this leads to synthetic lethality and cell death (Figure 3) [69].

#### 2.5.2. Homologous Recombination Repair (HRR) Gene Mutations

BRCA1 and BRCA2 are paramount in double-stranded DNA break repair via the homologous recombination repair (HRR) process. *BRCA1/2* mutation status may have implications on treatment selection. Loss of *BRCA* results in homologous recombination deficiency, rendering cells sensitive to platinum chemotherapy as well as to inhibitors of the DNA repair enzyme PARP. In mCRPC, approximately 15–20% of patients have genetic changes in HRR genes, which has led to the approval of PARP inhibitors in these patients [70].

Olaparib was studied for use in patients who have progressed on enzalutamide or abiraterone and have a deleterious germline or somatic mutation in genes involved in HRR, including the genes BRCA1, BRCA2, ATM, CHEK1, CHEK2, PALB2, RAD51B, RAD51C, RAD51D, RAD54L, BARD1, BRIP1, CDK12, or FANCL [71,72]. Patients were randomized to receive olaparib or the physician’s choice of either enzalutamide or abiraterone. In patients with BRCA1, BRCA2, or ATM mutations, both PFS and OS were significantly longer with a PFS of 7.4 months vs. 3.6 months (hazard ratio for progression or death, 0.34; 95% confidence interval, 0.25 to 0.47; *p* < 0.001), and OS of 19.4 months vs. 14.7 (hazard ratio for death, 0.69; 95% confidence interval [CI], 0.50 to 0.97; *p* = 0.02) with olaparib compared to enzalutamide or abiraterone. A significant improvement in rPFS was also seen when combining results with a cohort of patients with other HRR defects. Olaparib is also being investigated in ongoing studies in earlier stages of mCRPC in combination with ARAT treatment [26].

Rucaparib, on the other hand, is Food and Drug Administration (FDA) approved for patients with alterations in BRCA1 or BRCA2 who have progressed on ARAT treatment as well as taxane chemotherapy [73]. Germline or somatic mutations in BRCA1 and BRCA2 from the tumor or liquid biopsy are now standardly tested as a predictive biomarker for the use of PARP inhibitors in patients with mCRPC in the second line [71,73,74]. Other PARP inhibitors, such as talazoparib and niraparib, are also being investigated [75,76]. Interestingly, there seems to be a class effect whereby PARP inhibitors seem to have increased activity in patients with BRCA2 mutations compared to BRCA1 [77,78]. A meta-analysis of PARP inhibitors in mCRPC demonstrated that BRCA mutations and HRR mutations were effective biomarkers in predicting response to PARP inhibitors in mCRPC [79].

The American College of Medical Genetics and Genomics (ACMG) guidelines recommend germline testing in patients with ≥3 first-degree relatives or ≥2 first-degree relatives < 55 years of age diagnosed with prostate cancer or patients present with high-risk prostate cancer (Gleason score > 7), or there is a family history of two individuals with breast, ovarian, or pancreatic cancers [80]. Similarly, National Comprehensive Cancer Network (NCCN) guidelines recommend germline testing for patients with a strong family history of prostate cancer, high-risk or very high-risk localized prostate cancer, or metastatic prostate cancer regardless of family history, intraductal histology, or Ashkenazi Jewish ancestry [81]. Despite this, there are challenges in obtaining testing for eligible individuals. Genetic testing for HRR genes can be costly, especially with NGS panels that include sequencing of multiple genes at once [82]. There are also limited resources for genetic counseling, which currently limits the availability of PARP inhibitor treatment worldwide [82].

#### 2.5.3. PI3K/AKT/mTOR

Phosphatase and tensin homolog (PTEN) is a tumor suppressor gene involved in the phosphatidylinositol-3-kinase (PI3K)/AKT signaling pathway. Approximately 50% of CRPC harbors inactivating mutations in PTEN, which are a poor prognostic biomarker [83]. Mutations in this pathway are associated with resistance to AR-targeting treatments [84]. Inactivation in PTEN results in activation of the PI3K/AKT pathway, which leads to downstream promotion of growth factors and tumor proliferation [85]. The PI3K/AKT pathway and the AR pathways are inversely related, whereby activation of one pathway leads to the suppression of the other [84,86]. Conversely, inhibiting one pathway leads to activation of the other. Thus, it explains why the PI3K/AKT pathway would be activated as a mechanism of resistance to chronic blockade of the androgen receptor pathway with ADT and ARATs. Inactivation of PTEN is associated with resistance to ARATs such as abiraterone [86].

A number of PI3K, mTOR, and AKT inhibitors were investigated for the treatment of mCRPC. One systematic review did not show the efficacy of PI3K or mTOR inhibitors, but AKT inhibitors were promising [87]. Ipatasertib is an AKT inhibitor that is currently being investigated in the treatment of mCRPC in combination with abiraterone, with the idea that inhibiting both pathways at once could overcome resistance and allow for improved efficacy of treatment (Figure 1B) [88]. Indeed, this phase III trial examining pimasertib in combination with abiraterone and prednisolone in mCRPC resulted in improved rPFS (16.5 months versus 18.5 months, hazard ratio 0.77, CI 0.61–0.98, *p* = 0.034) and ORR compared to abiraterone and prednisolone and placebo in patients with PTEN loss mutations, although OS data are immature [88]. Another AKT inhibitor, capivasertib, was investigated in combination with docetaxel compared to docetaxel alone in a phase II study. Although the primary endpoint of PFS was not met, the secondary endpoint of OS was longer in the combination arm, and further studies are warranted to identify which patients could potentially benefit from this combination [89]. Capivasertib, in combination with enzalutamide, is also being investigated in a phase I trial [90]. Furthermore, AKT inhibitors may be showing promise in the treatment of mCRPC with acquired resistance to PARP inhibitor monotherapy and are being investigated in combination with PARP inhibitors [5].

Aurora kinase inhibitor alisertib was investigated in a phase II trial for the treatment of patients with metastatic castrate-resistant NEPC [91]. Although the trial did not meet its endpoint for PFS, there were some impressive responses in patients with tumors with N-myc and Aurora-A overactivity [91].

### 2.6. Immunotherapy

#### 2.6.1. Immune Checkpoint Inhibitors

**Figure 4:**
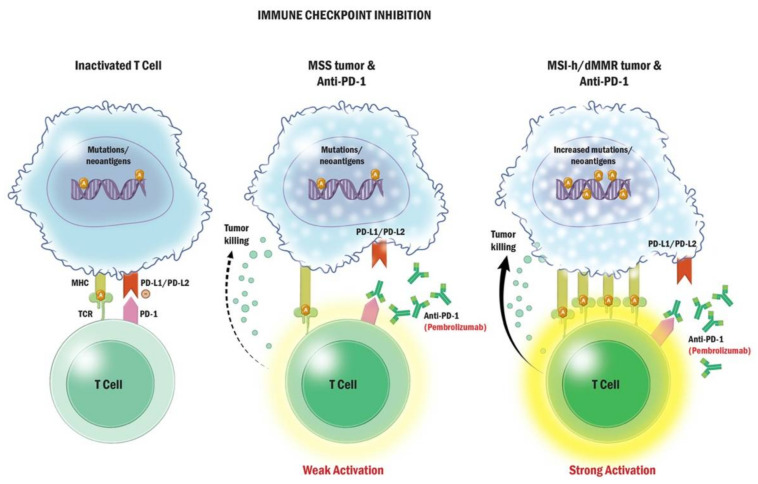
Mechanism of Action of Immunotherapy. Tumors highly express PD-L1 on their cell surface as a mechanism of camouflaging themselves from attack from the immune system. When PD-1 on T cells binds to its ligand PD-L1 on tumor cells, it sends an inhibitory signal to the T-cell, disguising itself as healthy tissue. In the presence of PD-1 checkpoint inhibitors, this interaction is blocked, allowing for T cell activation and recognition of the tumor cell as foreign, resulting in cytotoxic cell death. When tumors have high mutational burdens (TMB) or high microsatellite instability (MSI-H), they generate more neoantigen protein strands from mutations on the DNA strands (represented by an orange circle with the letter “a”). These antigens are then presented to the T cell resulting in stronger activation of T cell in the presence of PD-1 inhibitors.

Immune checkpoints are self-recognition proteins that function to suppress the immune response to prevent tissue damage to the host in response to inflammation [92]. Although immune checkpoint inhibitors have been revolutionary in the treatment of other types of cancer, such as melanoma and non-small-cell lung cancer, they have not been as effective in prostate cancer. This is to some extent because prostate cancer is an immunologically cold tumor. CTLA-4 is an immune checkpoint receptor homolog to cluster of differentiation (CD) 28 and is expressed on T-cells. CTLA-4 inhibitors, such as ipilimumab, increase T-cell activation and infiltration into tumors [92]. PD-1, on the other hand, is a co-signaling receptor part of the B7/CD28 family, expressed on activated T-cells, B-cells, natural killer cells, and exhausted T-cells. Its ligand, PD-L1, is highly expressed on some tumor cells as a mechanism of evading the host’s immune system. When bound to PD-L1, PD-1 attenuates T-cell receptor signaling, reducing T-cells’ activity and effector functions in peripheral tissues [92]. Blocking this interaction allows the resumption of T-cell activity in the periphery (Figure 4).

While immune checkpoints have a role in protecting host tissue from an autoimmune response, tumors have exploited these mechanisms as a way to camouflage from the immune system by disguising themselves as ‘self’ through increased expression of PD-L1 on their surface, allowing them to escape immune surveillance [93].

##### PDL-1/TMB/MSI-H/dMMR

The ipilimumab/nivolumab combination was explored in patients with mCRPC previously treated with either an ARAT pre-chemotherapy or after treatment with taxane chemotherapy in the phase II CheckMate 650 study [94]. Preliminary results showed two patients in each of the cohorts had complete responses, and ORR were 25% and 10%, respectively, with a median OS of 19.0 and 15.2 months [94]. In an exploratory analysis of those patients with available exome sequencing data with tumor mutational burden (TMB) above the 50th percentile, the response rates were 50.0% vs. 5.3%, rPFS was 7.4 vs. 2.4 months, and median OS was 19.0 vs. 10.1 months [94]. Similarly, patients with HRR-deficient tumors had response rates of 50.0% compared to 22.6%, median rPFS of 7.3 vs. 4.4 months, and median OS was not reached versus 19 months [94]. Among patients with available PD-L1 expression status, those with PD-L1 ≥ 1% vs. those with PD-L1 < 1% had an ORR of 36.4% vs. 12.1%, median rPFS of 5.6 vs. 3.9 months, and OS not reached vs. 15.2 months [94]. These early data are promising for a potential future role for combination immune checkpoint inhibition in the treatment of refractory mCRPC in select patients with above-median TMB, PD-L1 expression of 1% or greater, or deletions in HRR repair genes.

Anti-PD-1 therapy with pembrolizumab was also explored in pre-treated mCRPC patients in the KEYNOTE-199 study, but response rates were unimpressive regardless of PD-L1 status, with less than 5% response rate in either cohort, although those who did respond sometimes had durable response [95]. Despite this, patients with prostate cancer may qualify for treatment with pembrolizumab under the tissue-agnostic food and drug administration (FDA) approval in patients with mCRPC with high levels of microsatellite instability (MSI-H) and mismatch repair deficiency (dMMR) due to mutations in MLH, MSH2, MSH6, and PMS2 genes [96]. Mutations in these genes lead to a high number of somatic mutations compared to MMR-proficient tumors, including mutations in repetitive sequences of DNA called microsatellites. MSI-H status in tumors often leads to a larger number of DNA mutations and productions of neoantigens, which are then presented to T-cells via major histocompatibility complexes on the tumor cell, and thus higher levels of infiltrating lymphocytes [96]. With more antigen presentation and lymphocyte infiltration, PD-1 inhibition with pembrolizumab becomes more potent, leading to the higher activity of the T-cell tumor killing (Figure 4) [96].

Interestingly, however, the patients in KEYNOTE-199 with mCRPC who responded to pembrolizumab were not MSI-H, suggesting there may be patients who could potentially respond to immunotherapy who are not identified by using MSI-H alone as a predictive biomarker [95]. Inactivating mutations in cyclin-dependent kinase CDK12 are present in up to 7% of mCRPC tumors and were also associated with response to immune checkpoint inhibition in mCRPC [97]. A phase II trial of ipilimumab and nivolumab in patients with tumors harboring CDK12 mutations is currently underway (NCT03570619) [98]. Another phase II trial investigated the ipilimumab and nivolumab combination in mCRPC patients with AR-V7 mutation and showed that those with mutations in DNA repair genes had more favorable biochemical and radiographic responses [99]. Given that tumors with mutations in DNA repair genes also seem to respond to PARP inhibitors, the combination of PARP inhibitors with immune checkpoint inhibitors is being investigated (NCT02484404) [100].

### 2.7. Vaccine Treatments

#### 2.7.1. Prostatic Acid Phosphatase

Therapeutic cancer vaccines were tested in prostate cancer through the combination of viruses expressing antigens specific to prostate tissue in order to induce an immune response directed at the prostate cancer tissue. Sipuleucel-T is a dendritic cell vaccine treatment approved for use in the United States for mCRPC, which is a fusion protein made from a patient’s own peripheral blood mononuclear cells and consists of a fusion of a common prostate cancer antigen (prostatic acid phosphatase) linked to granulocyte-macrophage-colony-stimulating factor [101]. The treatment induces CD4+ and CD8+ T-cell activity against the tumor antigen [101]. It remains the only currently approved vaccine therapy for prostate cancer but has not been widely adopted worldwide due to its complex administration, high cost, and limited manufacturing capacity [102]. Another dendritic cell vaccine activated with a prostate cancer natural killer cell is currently in advanced phase III research [103].

#### 2.7.2. Prostate-Specific Antigen (PSA)

PROSTVAC is a recombinant poxvirus that expresses PSA with an immune-enhancing costimulatory molecule, stimulating the production of an immune response against tissue expressing PSA [102]. It was investigated in combination with CTLA-4 inhibition [104]. Although it showed promise in phase II studies, a phase III trial later showed no effect on OS and PFS in mCRPC patients [105,106]. Another vaccine treatment for prostate cancer called GVAX, consisting of prostate cancer cell lines transfected with a human colony-stimulating factor gene, was examined in two phase III trials (VITAL 1 and 2), which were discontinued due to futility [102,107]. However, GVAX was also investigated in combination with CTLA-4 blockade in an early phase I–II trial in chemotherapy-naive mCRPC patients and showed reasonable anti-tumor activity [108].

## 3. Limitations

Although molecular analyses are allowing a movement toward precision oncology, there are several limitations to the use of predictive biomarkers currently. Some biomarkers, such as dMMR genes or PDL-1 expression, have heterogeneity in expression levels due to the use of different lab assays, making standardization difficult [109,110,111,112]. PDL-1 expression also varies in predictive value among different types of tumors, and some patients respond to immunotherapy despite the lack of PDL-1 expression [107]. Some somatic mutations may be acquired throughout the course of treatment, and sampling from the initial biopsy at diagnosis may not be reflective of the current biology of the tumor as it has evolved over time. Even within a single individual, there can be heterogeneity in somatic mutations in sampling from different metastatic sites [113]. The sample can be many years old from the original biopsy, with limited viable samples available for testing. However, new diagnostic tissue at diagnosis of metastatic disease can also be difficult to obtain, given the most common metastatic site is bone [82]. Furthermore, molecular analysis can be costly, and identifying heritable mutations should ideally be accompanied by genetic counseling, which limits the widespread utilization of biomarker-driven therapy worldwide.

Sampling genetic material can furthermore be a challenge. Blood samples only identify germline mutations in BRCA, for example, whereas tumor or tissue samples can test both somatic and germline mutations [114]. Germline mutations are estimated to occur in approximately 12% of men with metastatic prostate cancer, whereas somatic mutations can be found in over 30% of men with metastatic prostate cancer [115,116]. This could result in missing an opportunity to treat 15–20% of patients who may have a somatic mutation but not a germline one. Blood samples from ctDNA also are less sensitive than tissue samples and, in some tumor sites, have a concordance of 80% with tissue samples [82]. A wide array of assays are utilized across laboratories, making standardization in terms of specificity and sensitivity difficult. Furthermore, tumor sampling does not distinguish between germline or somatic mutations, which of course, can have important implications for at-risk relatives and genetic counseling [82]. All of the above highlight some of the difficulties with putting biomarker-driven treatment into clinical practice and the need for the development of improved access worldwide and cost-effective testing strategies.

Many of the biomarkers discussed are not entirely sensitive or specific enough to be relied on completely, but nonetheless can still be helpful in treatment selection. It is possible that a panel of biomarkers could be utilized to help overcome this issue [117]. New biomarkers, such as mutations in genes involved in the MYC pathways and therapeutic targeting of epigenetic changes with the use of bromodomain and extra-terminal domain (BET) inhibitors, are also being investigated in early phase I trials [118].

As was discussed throughout this paper, a number of these oncogenic drivers are interrelated, and inhibition of one pathway can lead to acquired resistance by attenuation or activation of another pathway. Thus, combination strategies with therapies with differing mechanisms of action may be warranted to overcome the development of resistance to single-agent treatment [117,119].

PEACE-1 and ARASENS are recent trials that have found survival benefits to combining ARATs with chemotherapy in the mCSPC setting [120,121]. Novel agents, such as AR-V7 direct antagonists, were also examined in combination with ARATs as a mechanism for overcoming acquired resistance to ARATs. Niclosamide is being tested in combination with enzalutamide (NCT0312978) and in combination with abiraterone and prednisone (NCT02807805) [122].

PARP inhibitors have also recently been investigated in studies in combination with ARATs. In PROPEL III, patients were randomized in a double-blinded, placebo-controlled trial to receive abiraterone and prednisone/prednisolone plus olaparib or placebo in the first-line setting in patients with mCRPC regardless of HRR status by ctDNA [123]. The results showed a rPFS benefit to the addition of Olaparib compared to abiraterone alone (24.8 vs. 16.6 months; hazard ratio [HR] 0.66, 95% confidence interval [CI] 0.54–0.81; *p* < 0.0001). Although the results based on subgroup analyses were more profound in those with HRR mutations (HR 0.54, 95% CI 0.36–0.79), there was still benefit in patients without HRR mutations (HR 0.76, 95% CI 0.59–0.97). There was also a trend towards improved overall survival, although data are not yet mature. Similar results were presented from the MAGNITUDE phase III trial comparing abiraterone and prednisone/prednisolone plus either niraparib or placebo [124]. Results showed improvement in rPFS in the treatment arm in patients with HRR mutations.

## 4. Conclusions

Treatment of advanced prostate cancer has drastically changed over the last decade, with a number of new treatments, including ARATs, PARP inhibitors, theragnostic, immunotherapy, and other targeted agents showing efficacy and even obtaining FDA or Health-Canada approval for use, not to mention funding for testing by third-party payers such as insurance companies or government funding. Although there are a large number of treatment options available with varying mechanisms, identifying optimal treatment sequencing to maximize the chance of response while minimizing toxicity has been lacking, and there is an increasing need for predictive biomarkers to allow for more personalized medicine. Emerging capability for molecular analysis with whole-genome sequencing and next-generation sequencing for detection of CTCs and ctDNA resulted in the identification of an important number of potentially predictive biomarkers in prostate cancer. Although this kind of analysis can be costly and challenging to obtain tissue for analysis, liquid biopsies allow for more widespread utility due to the ease of obtaining samples. Predictive biomarkers are often still somewhat limited in that they often lack sensitivity and specificity, but a panel of biomarkers or further research may improve our ability to hone in on specific individualized treatments.

In summary, there is a need to address the heterogeneity of the disease with a more refined approach, given the direction toward more personalized therapies in prostate cancer. That would require not only more comprehensive and wider biomarker-driven targets but also an accessible panel of biomarkers to allow for universal use.

A number of potential biomarkers were discussed in this review, along with their associated biomarker-driven treatment. Table A1 lists predictive biomarkers that were identified in prostate cancer and may be helpful in personalizing treatment decisions for patients.

## Appendix A

**Table A1:** Predictive Biomarkers for Treatments of Advanced Prostate Cancer.

Type of Treatment	Treatment Name	Predictive Biomarker
ARATs	Abiraterone and prednisone Enzalutamide	Neutrophil-to-lymphocyte ratio [27] Serum Testosterone [29] AR-V 7 [31,32,33,125] CTCs and ctDNA [8,31,32,33]
Chemotherapy	Taxanes	Neutrophil-to-lymphocyte ratio [27]
		Serum Testosterone [29]
		ERG/SOX9 [45,46]
	Platinums	DNA Damage repair gene alterations [51]
		SLFN11 expression [52]
PSMA-directed therapy	177-Lu-PSMA-617	PSMA expression [61,65,105]
	BiTE immunotherapy	
	CAR-T	
Targeted Therapies	PARP inhibitors	HRR mutations [71,73,74]
	AKT Inhibitors	PTEN loss [88]
Immunotherapy	CTLA-4 inhibitor/PD-1 inhibitor	HRR mutations [94]
		TMB [94]
		PDL-1 expression [94]
	PD-1 inhibitor	PDL-1 expression
		MSI [96]
		dMMR [96]
Vaccines	Sipuleucel-T	Prostatic acid phosphatase [101]
	PROSTVAC	PSA [105,106]

Abbreviations: AR = androgen receptor; ARATs = androgen receptor-axis targeted therapies; BiTE = bispecific T-cell engager; dMMR = deficient mismatch repair; CTLA-4 = cytotoxic T-lymphocyte associated antigen-4; DNA = deoxyribonucleic acid; HRR = homologous recombination repair; LHRH = luteinizing hormone-releasing hormone, MSI = microsatellite instability; PARP = poly adenosine diphosphate-ribose polymerase; PD-1 = programmed cell death protein 1; PD-L1 = Programmed cell death ligand-1; PSA = prostate specific antigen; PSMA = prostate-specific membrane antigen; RANK = Receptor activator of nuclear factor kappa-B ligand; TMB = tumor mutational burden.
